# Integrative identification of insect flower visitors associated with avocado flowering in Western Australia

**DOI:** 10.1093/ee/nvag067

**Published:** 2026-06-18

**Authors:** Miyuki Taniguchi, Giles Hardy, Wei Xu

**Affiliations:** School of Agricultural Sciences, Murdoch University, Murdoch, WA, Australia; Food Futures Institute, Murdoch University, Murdoch, WA, Australia; Harry Butler Institute, Murdoch University, Murdoch, WA, Australia; ArborCarbon Pty Ltd. Rota Trans 1, Murdoch University, Murdoch, WA, Australia; School of Agricultural Sciences, Murdoch University, Murdoch, WA, Australia; Food Futures Institute, Murdoch University, Murdoch, WA, Australia

**Keywords:** DNA barcoding, *Lasioglossum*, *Simosyrphus grandicornis*, avocado flower visitor, taxonomy

## Abstract

Accurate identification of insect flower visitors is fundamental for understanding crop pollination systems and supporting sustainable production. In Western Australia, avocado (*Persea americana* Mill.) production relies heavily on insect visitation, yet the taxonomic identity of native flower-visiting taxa remains poorly resolved. This study provides the first integrative identification of insects associated with avocado flowering in Western Australia using a combination of morphological examination and mitochondrial cytochrome c oxidase subunit I (COI) DNA barcoding. Insects were collected from commercial avocado orchards in Carabooda and Pemberton during the flowering seasons of 2019–2021. Besides honeybees, morphological assessment identified 5 dominant native bee taxa, primarily within *Lasioglossum* (Halictidae), and 3 hoverfly taxa (Syrphidae). DNA barcoding confirmed species-level identities for *Lasioglossum lanarium* (Smith), *L. castor* (Smith), and the syrphids *Simosyrphus grandicornis* (Macquart) and *Eristalis tenax* (Lannaeus) (≥99% to 100% sequence similarity). In contrast, COI sequences from 3 additional *Lasioglossum* lineages showed lower similarity to available reference records (eg 96%, 91%, and 94%) and are therefore treated conservatively as *Lasioglossum* sp. 3 to 5 (morphospecies) rather than assigned species-level names. Overall, 73% of putative avocado flower-visitor taxa were shared between regions, indicating a broadly consistent core assemblage across contrasting climatic zones. By clarifying the taxonomic identity and reporting confidence of key avocado-associated taxa, this study provides a baseline for future work evaluating visitation dynamics, pollen transfer, and management strategies in Australian avocado orchards.

## Introduction

Avocado (*Persea americana* Mill.) relies heavily on insect visitation for fruit set because of its protogynous dichogamy and complementary flowering types (Davenport 1986, Sedgley 1987, Ish-Am and Eisikowitch 1993). A diverse assemblage of flower-visiting insects, including managed honeybees and native taxa, has been recorded in avocado systems worldwide (Klein et al. 2007, Potts et al. 2010, Genung et al. 2017). Over the past decade, Australian avocado production increased from 48,715 tons in 2013/2014 (Avocados Australia 2022) to 150,913 tons in 2023/2024 (Avocados Australia 2024). In 2023/2024, Western Australia (WA) produced 65,801 tons, accounting for 44% of national production (Avocados Australia 2024), underscoring the importance of understanding insect visitation in Western Australian orchards. The 2 main avocado-growing regions of WA also differ in climatic conditions and flowering phenology, with Carabooda flowering earlier (spring) and Pemberton flowering later in the season, providing a useful contrast for documenting avocado flower-visitor assemblages.

Despite the scale of production, the taxonomic composition of insects visiting avocado flowers in WA remains poorly resolved. Because avocado fruit set depends on insect-mediated pollen transfer, identifying which taxa visit flowers (and contact reproductive structures) is essential for interpreting visitation patterns and informing pollination management in orchards. Most previous work has focused primarily on *Apis mellifera*, with comparatively limited attention given to native taxa that may contribute to flower visitation and potentially complement managed honeybees (Rader et al. 2016). Recent Australian work has also highlighted the potential contribution of Diptera to avocado pollination, including syrphids and calliphorid flies under commercial conditions (Cook et al. 2023, 2025). More broadly, multi-region studies of Australian avocado systems show that non-bee visitor groups, including Diptera such as Syrphidae and Calliphoridae, can be recurrent components of avocado flower-visitor assemblages across regions and years (Willcox et al. 2019). Robust taxonomic identification is therefore required to characterize the composition of avocado flower-visitor communities and to enable meaningful comparisons among orchards and regions. This need is particularly relevant in WA, where climate change, habitat fragmentation, and agricultural intensification may further affect native insect communities and the services they provide (Cook et al. 2007, Batley and Hogendoorn 2009).

Accurate identification underpins ecological interpretation of flower-visitor assemblages because it provides the basis for comparing taxa across sites and for linking species presence to hypotheses about visitation behavior and pollen transfer. Morphological identification remains indispensable for recognizing taxa and assessing traits relevant to flower visitation (Michener 2007), yet diagnostic characters can be difficult to interpret among small-bodied or morphologically similar species. DNA barcoding using the mitochondrial cytochrome c oxidase subunit I (COI) gene has therefore emerged as a valuable complementary tool for resolving ambiguous identifications and supporting transparent reporting of identification confidence (Hebert et al. 2003, Schmidt et al. 2015, Gibbs 2018). Integrating morphological and molecular data (integrative taxonomy) can improve resolution, reveal cryptic diversity, and strengthen biodiversity assessments when reference coverage is sufficient (Magnacca and Brown 2012).

Among native flower visitors, bees of the genus *Lasioglossum* (Halictidae) are abundant and diverse in WA. These solitary or primitively social bees exhibit variation in nesting behavior, foraging ecology, and morphology (Walker 1995, Gibbs 2010, Jackson et al. 2025). Many *Lasioglossum* species are sensitive to environmental stressors, including pesticide exposure, habitat degradation, and pathogens (Müller et al. 2006, Knauer et al. 2022), emphasizing their ecological importance and vulnerability. However, species-level identification can be challenging because interspecific morphological differences may be subtle, and reference sequences are incomplete for parts of the Australian fauna. Accurate identification and documentation of *Lasioglossum* species are therefore essential for interpreting patterns in avocado flower-visitor assemblages and for enabling meaningful comparisons among orchards and regions. Hoverflies (Diptera: Syrphidae) represent another important group of flower visitors. Adult syrphids feed on pollen and nectar, while larvae occupy diverse niches and often act as predators of soft-bodied pests such as aphids (Finch and Cook 2020). Although more than 6,000 syrphid species have been described globally, aspects of their taxonomy and evolutionary relationships remain unresolved, and their diversity in Australian orchard systems remains incompletely documented (Dunn et al. 2020). In Australian orchards, *Simosyrphus grandicornis*, *Melangyna viridiceps*, and *Eristalis tenax* are among the most frequently observed floral visitors (Dunn et al. 2020), yet their occurrence and identification in WA avocado orchards have not been comprehensively assessed.

This study applies an integrative identification framework to characterize dominant bee and hoverfly flower visitors associated with avocado flowering in 2 contrasting regions of WA: Carabooda (subtropical) and Pemberton (temperate). Putative flower-visitor status was defined operationally as contact with floral reproductive structures during direct observations. Specifically, we aimed to (i) refine taxonomic identification (to the lowest reliable rank) of major avocado flower-visiting taxa using COI DNA barcoding in combination with morphology, (ii) assess whether barcoding reveals divergent *Lasioglossum* lineages indicative of incomplete reference coverage and/or cryptic diversity, and (iii) generate reference COI sequences and associated metadata to support future ecological studies of avocado flower-visitor communities in Australian agroecosystems. By establishing a robust and transparent taxonomic baseline, this study provides a foundation for subsequent work evaluating visitation dynamics, pollen transfer, and management options in WA avocado orchards.

## Materials and Methods

### Study Regions and Orchards

**Fig. 1.**
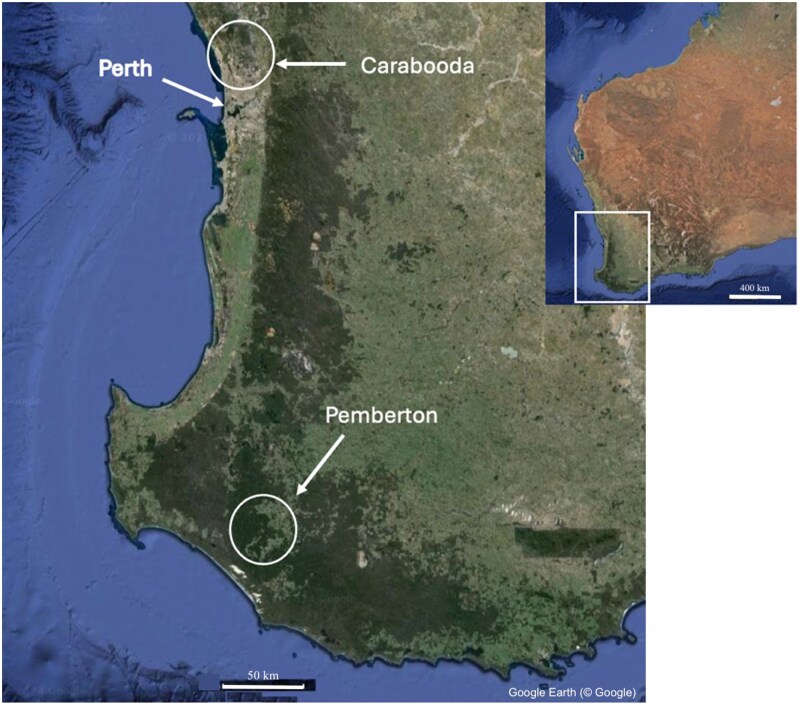
Study regions and avocado orchard sampling sites in Western Australia. Maps show the 2 non-overlapping flowering regions surveyed, Carabooda (Metropolitan Perth region) and Pemberton (Southwest), where avocado flower-visiting insects were sampled during the 2019–2021 flowering seasons. Site locations correspond to orchards sampled for direct observations and passive trapping (pan and vane traps), and points indicate orchard sampling areas within each region. Base map imagery from Google Earth.

Fieldwork was conducted in 2 major avocado-producing regions of WA with non-overlapping flowering periods: Carabooda (−31.605, 115.714; Metropolitan Perth) and Pemberton (−34.441, 116.048; Southwest) (Fig. 1). Both regions grow the cultivar ‘Hass’, which accounts for >95% of avocado production in WA (McCarthy 2001). Managed *Apis mellifera* hives were deployed at approximately 2 hives per hectare following standard industry practice.

The Carabooda orchard is located ∼40 km north of Perth and surrounded by mixed agricultural land and remnant bushland. Sampling occurred from mid-September to late October (2019–2021) (Supplementary Fig. S1a). The Carabooda study orchard (AVOWEST) covers approximately 25 ha and is bordered by a plant nursery and pasture to the north and east, with another avocado orchard to the south, a vineyard, and remnant bushland to the west. Pemberton is located ∼320 km south of Perth and surrounded by native *Eucalyptus* forests. Surveys were conducted from late October to late November at Delroy Orchard (∼50 ha) in 2019 and West Pemberton Avocado Orchard (∼50 ha) in 2020–2021 (Supplementary Fig. S1b); these orchards are surrounded by jarrah and karri forest, with West Pemberton also adjacent to other avocado orchards and a reservoir to the south. The regions were selected because their flowering periods do not overlap, allowing temporal separation of sampling. The objective of this study was to characterize the composition of avocado-associated flower-visitor assemblages rather than to conduct direct temporal comparisons between regions; consequently, the non-overlapping flowering periods do not bias the taxonomic conclusions presented here. Sampling schedule and effort are summarized in Table 1.

**Table 1.** Sampling schedule and effort for visual observations and passive trapping in avocado orchards in Western Australia

Region (orchard area)	Year	Survey window(s)	Observation days	No. quadrat sessions (15 min)	Total observation time (h)	Vane traps (stations; traps total)	Vane deployment	Pan traps (stations; bowls total)	Pan deployment
**Carabooda (∼25 ha)**	2019	15 to 17 Oct; 20 to 25 Oct; 27 Oct	10	48	12.0	Not deployed	—	Not deployed	—
**Pemberton (∼50 ha)**	2019	20 to 22 Nov; 26 to 29 Nov	7	63	15.8	Not deployed	—	Not deployed	—
**Carabooda (∼25 ha)**	2020	12 to 18 Oct; 24 to 25 Oct	9	38	9.5	6 stations; 12 traps	∼14 d; contents collected every 3 to 5 d	12 stations; 24 bowls	Suitable weather days (∼8 AM to 4 PM) within survey window(s)
**Pemberton (∼50 ha)**	2020	19 to 23 Oct; 26 to 30 Oct	10	51	12.8	12 stations; 24 traps	∼14 d; contents collected every 3 to 5 d	24 stations; 48 bowls	Suitable weather days (∼8 AM to 4 PM) within survey window(s)
**Carabooda (∼25 ha)**	2021	1 to 6 Nov; 7 to 13 Nov	13	38	9.5	6 stations; 12 traps	∼14 d; contents collected every 3 to 5 d	12 stations; 24 bowls	Suitable weather days (∼8 AM to 4 PM) within survey window(s)
**Pemberton (∼50 ha)**	2021	14 to 19 Nov; 21 to 26 Nov	12	53	13.3	12 stations; 24 traps	∼14 d; contents collected every 3 to 5 d	24 stations; 48 bowls	Suitable weather days (∼8 AM to 4 PM) within survey window(s)

One quadrat observation session corresponds to a 15-min observation period (0.25 h). Traps were not deployed in 2019. Trap density was standardized between orchards by scaling the number of trap stations (paired blue/yellow units) to orchard area (Carabooda ∼25 ha; Pemberton ∼50 ha).

### Flower-visitor Sampling Design

Insect visitors to avocado flowers were assessed using a combination of direct floral observations and passive trapping. Direct observations were primarily conducted to confirm putative avocado flower-visitor status, defined as contact with floral reproductive structures during either the female or male flower phases, rather than to quantify visitation rates or pollination efficiency. Passive sampling using vane and pan traps was employed to characterize the broader orchard insect assemblage present during flowering and to obtain specimens for morphological identification and DNA barcoding. Sampling effort was standardized across regions and years to allow qualitative comparison of assemblage composition (Table 1). Standardization refers to using the same observation protocol (50 × 50 cm quadrat, 15-min sessions, comparable daily effort during peak flowering) and scaling trap stations to orchard area (Carabooda ∼25 ha; Pemberton ∼50 ha) to maintain comparable trap density. A summary of survey windows and effort (observation sessions and trap deployments) by region and year is provided in Table 1. Taxa observed contacting reproductive structures are referred to as putative avocado flower visitors, whereas taxa captured in traps but not observed on avocado flowers are treated as orchard-associated taxa and are not assigned pollinator status based on trap capture alone.

### Visual Observations

Direct floral observations were conducted to confirm putative avocado flower-visitor status, defined as contact with floral reproductive structures during female or male flower phases. A 50 × 50 cm handheld quadrat was placed on flowering branches (approximately 50% to 70% of flowers open) and observed for 15 min per quadrat. Two to 3 quadrats per tree were assessed at heights of 1 to 4 m, with observations conducted across 3 to 5 trees per sampling day. This quadrat-based approach was selected to standardize the observation area and duration across orchards while allowing close inspection of insect–flower interactions. Observations were designed to document flower-visitor presence and behavior rather than to quantify visitation rates. Insects that could not be reliably identified in the field were collected for subsequent laboratory identification. We acknowledge that visual observations may underestimate small, fast-moving, or cryptic insects and may be biased toward larger or more conspicuous taxa; accordingly, visual observations were complemented by passive trapping to better characterize the broader insect assemblage present within orchards.

### Passive Trapping

Passive sampling was conducted concurrently with observations during the 2020 and 2021 flowering seasons. Passive trapping was conducted in 2020 and 2021 only (no traps were deployed in 2019). Trap stations (paired blue/yellow units) were scaled to orchard area to maintain comparable density between regions: Carabooda used 6 vane-trap stations (12 traps total) and 12 pan-trap stations (24 bowls total), whereas Pemberton used 12 vane-trap stations (24 traps total) and 24 pan-trap stations (48 bowls total). Traps were deployed for approximately 2 wk per survey window, with vane-trap contents collected every 3 to 5 d and pan traps operated on suitable weather days during daylight hours.

Collected specimens were preserved in ethanol and stored at −20 °C until processing.

### Morphological Identification

Diagnostic characters including wing venation, antennal structure, scopal hair development, and body coloration were used to differentiate taxa (Halcroft and Batley 2014). Initial identifications were made to family and genus (and where possible subgenus) using general references for Australian insects and bees (Zborowski and Storey 2017, Houston 2018). Where species-level identification was attempted, taxon-specific revisionary keys were consulted (eg Walker 1995 for *Lasioglossum* [*Chilalictus*]). Final species assignments were reported conservatively and, for selected taxa, were supported by COI DNA barcoding. Native bee identifications were verified by Dr Terry Houston (Western Australian Museum). Syrphid flies were cross-checked using published diagnostic resources and online identification keys. Representative specimens were photographed and retained as voucher material in the Murdoch University Insect Collection.

### Molecular Identification

#### DNA Extraction and Barcoding

In Hymenoptera, species of the genus *Lasioglossum* are often difficult to identify morphologically, particularly by non-bee specialists; therefore, mitochondrial DNA barcoding targeting the COI gene was employed. The COI gene is widely used for species-level identification in insects because it is sufficiently conserved for primer universality while exhibiting interspecific divergence suitable for taxonomic discrimination (Hebert et al. 2003). In this study, COI-based identifications were interpreted using a conservative, evidence-based framework that considers both percent identity and Barcode Index Number (BIN) divergence on Barcode of Life Data Systems (BOLD) (Ratnasingham and Hebert 2013). In particular, BOLD BIN guidance was used as a heuristic indicator of conspecificity (typically <2.2% divergence) versus likely species-level separation (typically >4.4% divergence), with intermediate values treated as requiring additional evidence. Accordingly, species-level assignments were restricted to cases showing strong concordance between morphology and high-confidence COI matches, whereas lower-similarity matches were reported conservatively (eg as “cf.” taxa or provisional identifications) rather than as confirmed species records. We emphasize that COI similarity thresholds are heuristic and taxon-dependent; therefore, sequence identity values were interpreted conservatively, in conjunction with morphology, rather than as absolute species delimiters. Total genomic DNA was extracted from insect specimens using the DNeasy Blood and Tissue Kit (Qiagen, Hilden, Germany) following the manufacturer’s protocol. Polymerase chain reaction (PCR) amplification of a ∼650-bp fragment of the COI gene followed standardized protocols (Schmidt et al. 2015) using the universal primer pair LCO1490 (5′-GGTCAACAAATCATAAAGATATTGG-3′) and HCO2198 (5′-TAAACTTCAGGGTGACCAAAAAATCA-3′) (Folmer et al. 1994). Each 25 µl reaction contained 1× PCR buffer, 2.0 mM MgCl_2_, 200 µM of each dNTP, 0.2 µM of each primer, 0.5 U of Taq DNA polymerase (Invitrogen, United States), 0.4 mg ml^−1^ bovine serum albumin, and 1 µl of genomic DNA (5 to 50 ng). PCR amplification was performed on a thermal cycler (Veriti 96-Well Thermal Cycler, Applied Biosystems, United States) with the following conditions: initial denaturation at 95 °C for 2 min; 35 cycles of 95 °C for 30 s, 48 °C for 30 s, and 72 °C for 45 s; and a final extension at 72 °C for 5 min. PCR products were visualized on agarose gels and purified prior to sequencing. For samples that failed to amplify using universal primers, a bee-specific primer pair, BeeCox1F1 and BeeCox1R2 (Schmidt et al. 2015), was used either alone or in combination with the universal primers. The thermal cycling profile consisted of an initial denaturation step at 94 °C for 2 min, followed by 40 cycles of 94 °C for 30 s, 52 °C for 30 s, and 72 °C for 45 s. This was followed by a final extension at 72 °C for 10 min.

#### Sequence Analysis

Purified PCR products were sequenced using Sanger sequencing at the WA State Agricultural Biotechnology Centre (Murdoch University). Forward and reverse chromatograms were quality-checked, trimmed, and assembled into consensus sequences. Sequences were compared against the BOLD and NCBI BLAST databases to determine closest matches. Species-level identifications were assigned based on concordance between molecular matches and morphological traits. Chromatograms were inspected for ambiguous base calls and double peaks, and sequences were checked for unexpected indels or frameshifts prior to database matching.

## Results

### Insect Identifications

Insects from Halictidae, Apidae, Syrphidae, and Coccinellidae were directly observed visiting avocado flowers and recorded contacting reproductive structures; these taxa were therefore treated as putative avocado flower visitors (Table 2). Morphological identification was most challenging for Lasioglossum (Halictidae) and Syrphidae due to cryptic diversity and the limited availability of regional diagnostic keys. Passive trapping recorded multiple taxa in both Carabooda and Pemberton (Table 2). Across both regions, passive trapping collected 7,338 individuals during the 2020–2021 flowering seasons (Supplementary Table S1), providing a broad snapshot of the orchard insect assemblage present during flowering. Several focal taxa were recorded in both regions (Table 2), including *Lasioglossum lanarium* (COI-confirmed) and syrphid flower visitors; other records are reported at morphospecies or genus level where species-level confirmation was not supported. All taxa other than *Apis mellifera* were subjected to targeted morphological confirmation and/or COI DNA barcoding to refine identifications and document confidence in species assignments (Table 3).

**Table 2.** Insect taxa directly observed on avocado flowers in 2 Western Australian regions (Carabooda and Pemberton) during the 2019–2021 flowering seasons

Morphological ID	Molecular ID (COI/BOLD)	Carabooda	Pemberton	Evidence basis
***Lasioglossum lanarium***	*Lasioglossum lanarium*	✓	✓	Obs + traps
***Lasioglossum castor***	*Lasioglossum castor*	✓	–	Obs + traps
***Lasioglossum* sp. 3**	—	–	✓	Obs + traps
***Lasioglossum* sp. 4**	—	✓	✓	Obs + traps
***Lasioglossum* sp. 5**	—	–	✓	Obs
***Exoneura* sp. 1**	—	✓	–	Obs
***Exoneura* sp. 2**	—	–	✓	Obs
***Apis mellifera***	—	✓	✓	Obs + traps
***Simosyrphus grandicornis***	*Simosyrphus grandicornis*	✓	✓	Obs + traps
***Melangyna viridiceps***	—	✓	✓	Obs
***Eristalis tenax***	*Eristalis tenax*	✓	✓	Obs + traps
***Coelophora inaequalis***	—	✓	✓	Obs + traps
***Harmonia axyridis***	—	✓	✓	Obs + traps

Taxa listed were recorded contacting floral reproductive structures during observations and are treated here as putative avocado flower visitors. Passive trap captures (pan and vane traps,) indicate whether voucher specimens were also obtained for identification; trap capture alone was not used to assign flower-visiting status. Molecular IDs are reported only for taxa with COI results supporting species-level assignment under conservative criteria; other taxa are retained as morphospecies/genus-level IDs.

**Table 3.** Summary of COI DNA barcoding results for key avocado flower-visiting taxa from Carabooda and Pemberton, Western Australia

Taxon	*n* sequenced	Region(s)	COI length (bp)	Best match source	% identity	Interpretation
***Lasioglossum lanarium***	5	C + P	650	BOLD/GenBank	99 to 100	Confirmed
***L. castor***	3	C	633	BOLD/GenBank	99 to 100	Confirmed
***Lasioglossum* sp. 5 (cf. *hemichalceum*; BIN:AEC4092)**	3	P	616	BOLD/GenBank	94	cf.; not species-level
***Simosyrphus grandicornis***	5	C + P	∼540	BOLD/GenBank	100	Confirmed
***Eristalis tenax***	3	C + P	∼641	BOLD/GenBank	100	Confirmed

For each taxon, the table reports the number of specimens sequenced (*n*), COI fragment length, and the closest match in BOLD and/or GenBank with percentage identity. Species-level identifications are reported where sequence similarity supported reliable assignment, whereas lower-similarity matches are treated conservatively as cf. taxa or provisional identifications, reflecting incomplete reference barcode coverage for some Australian native bee lineages.

### Hymenoptera: Native Halictidae and Apidae Flower Visitors

#### *Lasioglossum* (Halictidae)

**Fig. 2.**
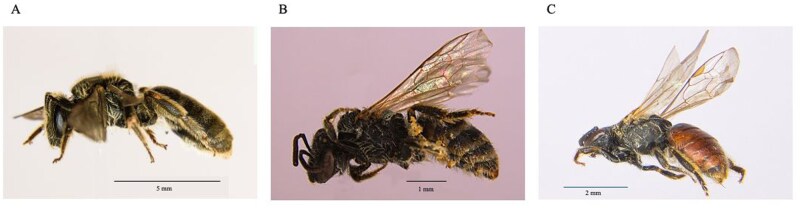
Representative females of 3 *Lasioglossum* (Halictidae) taxa recorded as avocado flower visitors in Western Australia. A) *Lasioglossum (Chilalictus) lanarium*; B) *Lasioglossum (Chilalictus) castor*; C) *Lasioglossum* sp. 5 (cf. *hemichalceum*; BIN:AEC4092). Taxa were identified using morphological examination and, where possible, confirmed by mitochondrial COI DNA barcoding (see Table 3 for COI match statistics and interpretive assignments). Images were captured using a Leica M165 FC stereo microscope. Scale bars as indicated.

Five *Lasioglossum* taxa were recorded as putative avocado flower visitors. Based on morphology supported by COI barcoding, *Lasioglossum lanarium* was confirmed in both regions, whereas *L.* castor was recorded only in Carabooda (Fig. 2; Table 3). In Pemberton, 3 additional *Lasioglossum* lineages were recovered but could not be assigned confidently to named species based on available reference coverage; these are therefore reported as *Lasioglossum* sp. 3 to 5 (Table 3). COI matches for these morphospecies were lower (sp. 3 = 96%, sp. 4 = 91%, sp. 5 = 94%); accordingly, they are treated conservatively and are not assigned species-level names. The 94% lineage is reported as *Lasioglossum* sp. 5, with the closest available BOLD BIN match (BIN:AEC4092; cf. *hemichalceum*) provided as contextual information only (Table 3). Overall, combined morphological and molecular evidence indicates that multiple *Lasioglossum* taxa are frequent avocado flower visitors, while highlighting that some lineages remain conservatively identified due to incomplete reference coverage (Table 3).

#### Apidae

**Fig. 3.**
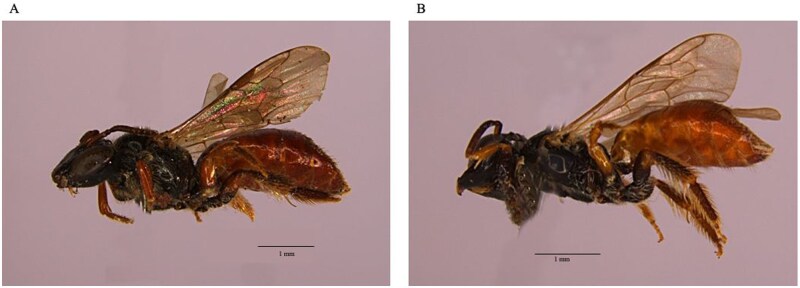
Representative Apidae taxa recorded as avocado flower visitors in Western Australia. A) *Exoneura* sp. 1 (female); B) *Exoneura* sp. 2 (female). Taxa were identified by morphological examination, and field observations were used to confirm avocado flower-visiting status. Images were captured using a Leica M165 FC stereo microscope. Scale bars as indicated.

Two *Exoneura* morphospecies (*Exoneura* sp. 1 and *Exoneura* sp. 2) and *Apis mellifera* were recorded as putative avocado flower visitors. *Exoneura* reed bees were common floral visitors in both regions, but species-level assignment during field observations was sometimes uncertain; consequently, some visitation records were conservatively retained at genus level (*Exoneura* spp.) when individuals could not be reliably distinguished on flowers. The *Exoneura* taxa were identified morphologically and were not subjected to COI barcoding in this study. Regionally, *Exoneura* sp. 1 was recorded only in Carabooda, whereas *Exoneura* sp. 2 occurred only in Pemberton (Fig. 3). *Apis mellifera* was abundant at both sites because managed hives were present; however, subsequent taxonomic resolution and molecular confirmation focused on native taxa.

### Diptera: Syrphidae Flower Visitors

**Fig. 4.**
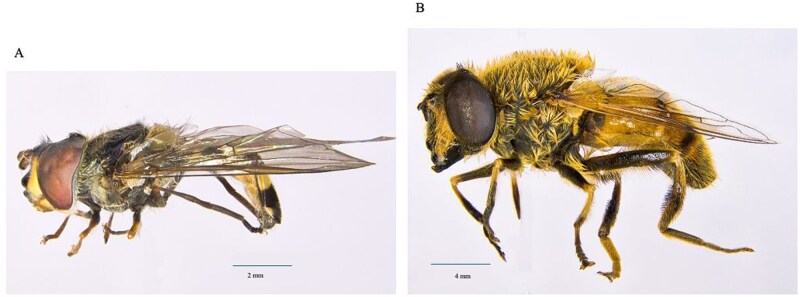
Representative syrphid taxa (Diptera: Syrphidae) recorded as avocado flower visitors in Western Australia. A) *Simosyrphus grandicornis* (adult); B) *Eristalis tenax* (adult). Taxa were identified by morphological examination and confirmed by mitochondrial COI DNA barcoding. Images were captured using a Leica M165 FC stereo microscope. Scale bars as indicated.

Three syrphid taxa were confirmed as avocado flower visitors in both regions: *Simosyrphus grandicornis*, *Melangyna viridiceps*, and *Eristalis tenax* (Fig. 4). Syrphidae represented the most abundant dipteran flower visitors recorded during the sampling period. Under field observation conditions, distinguishing *S. grandicornis* from *M. viridiceps* during visitation was difficult; therefore, some visitation records were pooled as *S. grandicornis* (functional group) to avoid overstating species-level certainty in observational data. COI barcoding confirmed the identities of *S. grandicornis* and *E. tenax*, with sequences showing 100% identity to reference sequences in BOLD/GenBank (Table 3).

### Other Orchard-Associated Taxa Captured by Passive Trapping

Across both regions, 7,338 insects were captured in vane and pan traps during the 2020–2021 flowering seasons. These captures summarize the broader orchard insect assemblage present during flowering and include both taxa also observed on avocado flowers and taxa not observed contacting avocado floral reproductive structures during timed observations. Several native bee taxa were captured but not observed visiting avocado flowers, including *Amegilla* spp., *Lipotriches* spp., additional *Lasioglossum* morphospecies, Colletidae, and Megachilidae (Supplementary Table S1; Supplementary Fig. S4). Notably, blue-banded bees (*Amegilla chlorocyanea* and *A. murrayensis*) were present in traps but were not observed actively foraging on avocado flowers during timed observations, suggesting that avocado was not a preferred floral resource during the sampling windows. DNA barcoding resolved several non-primary bee taxa, but confidence varied among groups depending on reference availability. For example, *Lipotriches (Austronomia) australica* and a *Megachile* specimen matched reference sequences. However, the *Megachile* sequence showed 94% identity to *M. chrysopyga* and is therefore treated conservatively as belonging to the *M. chrysopyga* species group rather than a confirmed species record. Within Colletidae, the specimen identified morphologically as *Trichocolletes* cf. *centrali* showed 89% COI similarity to *Trichocolletes* reference sequences, whereas the specimen identified morphologically as *Callomelitta antipodes* showed a 99.35% COI match to *C. antipodes*. These comparisons illustrate substantial variation in species-level resolvability among taxa due to uneven reference coverage (Supplementary Table S1).

## Discussion

### Main Findings and Regional Patterns

This study provides an integrative morphological and molecular assessment of insects associated with avocado flowering in WA. Using direct observations to document taxa contacting floral reproductive structures, together with passive trapping to characterize orchard assemblages and obtain voucher material, we identified a consistent set of key avocado flower visitors across 2 climatically distinct regions (Carabooda and Pemberton). More broadly, this pattern aligns with Australasian crop-survey work showing that a relatively consistent subset of insect groups can dominate flower-visitor assemblages, while taxonomic resolution remains a key constraint for interpreting ecological roles (Howlett et al. 2019). COI DNA barcoding provided strong confirmation for several taxa with good reference coverage (eg *Lasioglossum lanarium*, *Simosyrphus grandicornis*, *Eristalis tenax*), strengthening confidence in their occurrence in the study system. In contrast, several additional native bee lineages returned lower-similarity matches to available references, reinforcing that barcode coverage remains incomplete for parts of the Western Australian bee fauna. Collectively, these results show that an integrative approach can resolve dominant flower-associated taxa while also identifying cases where conservative reporting is warranted.

### Integrative Taxonomy: Added Resolution and Remaining Uncertainty

Morphological examination supported assignment of most specimens to genus, and in some cases to species, but species-level resolution was limited for small-bodied bees, particularly *Lasioglossum*, when assessed under routine survey conditions and in the absence of comprehensive regional diagnostic resources. COI barcoding therefore served as a critical verification layer and provided a transparent framework for reporting confidence.

For *Lasioglossum*, high-identity matches supported confident reporting for key taxa, whereas one lineage showed markedly lower similarity to reference sequences and is reported conservatively as a *Lasioglossum* cf. taxon rather than a confirmed species record. This pattern is consistent with incomplete reference representation and/or cryptic diversity within Australian halictids. For Syrphidae, barcoding helped confirm taxa that can be difficult to separate reliably during field observation. Overall, these findings support a 2-tier workflow for orchard pollinator surveys: morphology for initial sorting and diagnostic confirmation where reliable, and COI barcoding for verification and conservative handling of ambiguous lineages.

### Ecological Relevance and Management Implications

This study was designed to resolve taxonomic identity and document flower association, rather than to quantify pollination effectiveness. Nevertheless, establishing robust species- and lineage-level identities is a necessary prerequisite for future work linking taxa to pollen transport, visitation dynamics, and fruit set. The repeated detection of native bees and syrphid flies across regions indicates that Western Australian orchards support a diverse community of avocado flower visitors beyond managed honeybees. From an applied perspective, these results motivate management approaches that maintain habitat suitability for native insects during flowering (eg minimizing disruptive practices during peak bloom and reducing exposure risks from broad-spectrum insecticides), while recognizing that the contribution of individual taxa to avocado pollination requires targeted functional evaluation.

### Limitations and Next Steps

Two limitations are particularly relevant. First, COI-based identification depends on the completeness of reference libraries; lower-identity matches prevented species-level assignment for some native bee lineages, underscoring the need to expand curated barcode coverage for Western Australian taxa. Second, the combination of timed daytime observations and passive traps can underrepresent some flower visitors and overrepresent others; therefore, assemblage summaries should be interpreted as indicative rather than exhaustive.

Future work should combine taxon-resolved measures of visitation and pollen transport with expanded reference sequencing for underrepresented native bee groups and, where appropriate, spatially explicit habitat metrics (including remote-sensing approaches) (Willcox et al. 2018). This integrated evidence base will enable stronger inference about functional complementarity among managed and native flower visitors and support more robust orchard management recommendations.

## Supplementary Material

nvag067_Supplementary_Data

## Data Availability

Supporting materials, including the passive-trap taxon-group summary and representative diagnostic images, are provided in the Supplementary Material (Supplementary Table S1; Supplementary Figs S1–S4).
