# Supplementary figures and images for: Integrative identification of insect flower visitors associated with avocado flowering in Western Australia
Source: Environ Entomol. 2026 Jun 18;55(3):nvag067. doi: 10.1093/ee/nvag067 (PMC13278782; doi:10.1093/ee/nvag067)

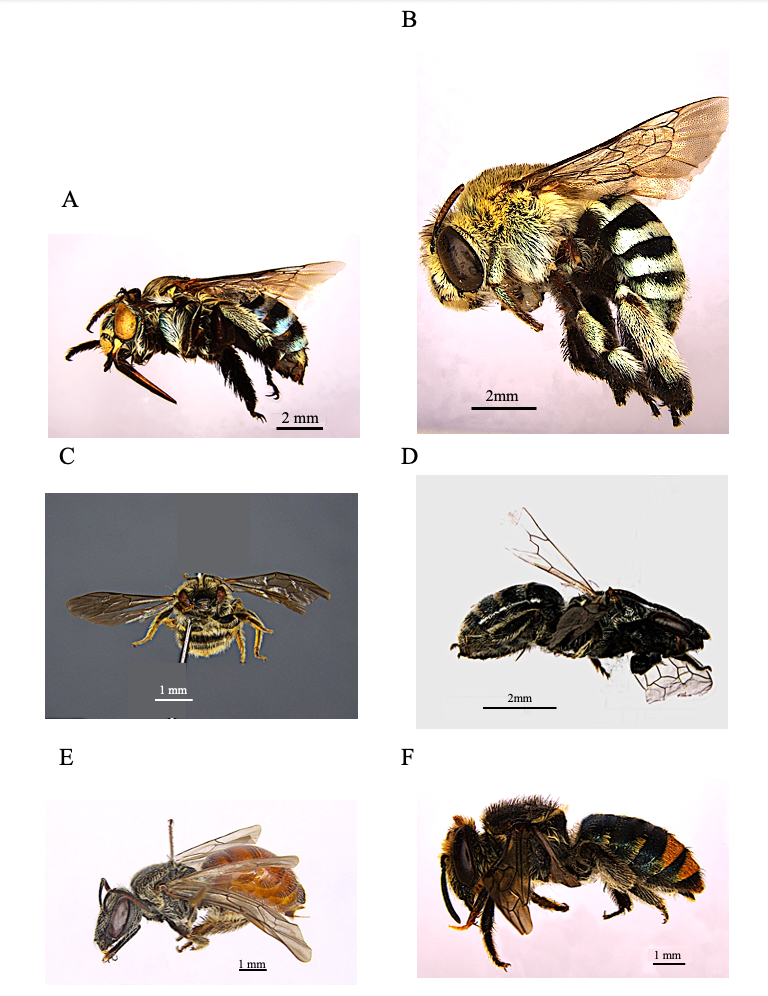

Supplement: nvag067_Supplementary_Data [file nvag067_supplementary_data.zip › Figure_S4-1.jpg]

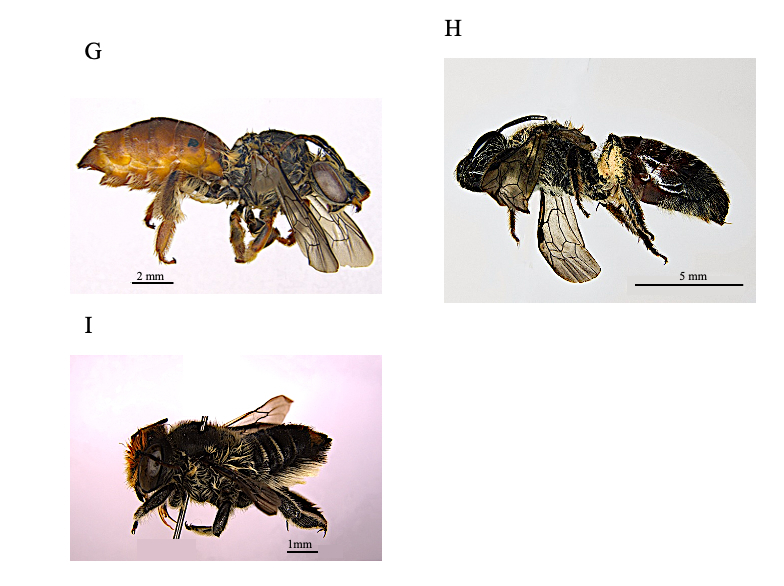

Supplement: nvag067_Supplementary_Data [file nvag067_supplementary_data.zip › 13-Jun-2026_063242_Figure_S4-2.jpg]
