# Supplementary material for: Integrative identification of insect flower visitors associated with avocado flowering in Western Australia
Source: Environ Entomol. 2026 Jun 18;55(3):nvag067. doi: 10.1093/ee/nvag067 (PMC13278782; doi:10.1093/ee/nvag067)
Supplement: nvag067_Supplementary_Data [file nvag067_supplementary_data.zip › Supplementary.zip!/Supplementary (Table S1 and Figure S1, S2, S3, $4).docx]

**Table S1.** Taxon-group summary of insects captured by passive trapping (pan and vane traps pooled) in avocado orchards in Western Australia during the 2020–2021 flowering seasons. Counts (n) are reported separately for Carabooda and Pemberton, with totals and percentages calculated from the combined trap catch (n = 7,338). Taxon groups are listed in the order of insect orders; “other” categories indicate specimens not assigned beyond broader groupings in the raw dataset.

| **Taxon Group** | **Carabooda**  **(n)** | **Pemberton**  **(n)** | **Total**  **(n)** | **% of Total** |  |
| --- | --- | --- | --- | --- | --- |
| *Apis mellifera* | 325 | 217 | 542 | 7.39 |  |
| Apidae | 98 | 52 | 150 | 2.04 |  |
| Halictidae | 239 | 805 | 1044 | 14.23 |  |
| Colletidae | 1 | 3 | 4 | 0.05 |  |
| Megachilidae | 10 | 4 | 14 | 0.19 |  |
| Hymenoptera other | 243 | 219 | 462 | 6.30 |  |
| Syrphidae | 576 | 162 | 738 | 10.06 |  |
| Drosophilidae | 34 | 150 | 184 | 2.51 |  |
| Diptera other | 1046 | 2045 | 3091 | 42.12 |  |
| Coleoptera other | 20 | 24 | 44 | 0.60 |  |
| Lepidoptera other | 165 | 33 | 198 | 2.70 |  |
| Hemiptera | 99 | 390 | 489 | 6.66 |  |
| Thysanoptera | 146 | 61 | 207 | 2.82 |  |
| Neuroptera | 114 | 35 | 149 | 2.03 |  |
| Insect other | 12 | 10 | 22 | 0.30 |  |
| **Total** | **3128** | **4210** | **7338** | **100.00** |  |

**Fig. S1**. Google Earth images showing the location and layout of the study orchards in Western Australia: (A) Carabooda; (B) Pemberton (a=Delroy: b= West Pemberton Avocado). Blue-outlined areas indicate the sections of the orchards where sampling was conducted (visual observations and/or trap deployments). Images were captured from Google Earth and annotated with site boundaries and scale bars.

**Fig. S2.** Key diagnostic characters used to identify *Lasioglossum* spp.: (A) female fifth metasomal tergite (T5) showing the median hair “furrow”; (B) forewing venation showing the three submarginal cells; and (C) scopal hairs on the hind leg used for assessing pollen-carrying structures.

**Fig. S3.** Key diagnostic characters of *Exoneura* spp.: (A) hind leg showing the reduced/absent scopal hairs on the tibia; (B) forewing venation with the anterior margin of the second submarginal cell extending at least to the first submarginal crossvein; and (C) forewing with the long Cu1 vein indicated by the arrow.

**Fig. S4.** Representative images of additional bee taxa collected by passive trapping in avocado orchards in Western Australia that were not observed contacting avocado floral reproductive structures during field observations. Panels show: (A) *Amegilla chlorocyanea*; (B) *Amegilla murrayensis*; (C) *Lasioglossum* sp. T1; (D) *Lasioglossum* sp. T2; (E) *Lasioglossum* sp. T3; (F) *Lipotriches* (*Austronomia*) *australica*; (G) *Trichocolletes* cf. *centrali*; (H) *Callomelitta antipodes*; (I) *Megachile* cf. *chrysopyga* (species group). Images were captured using a Leica M165 FC stereo microscope. Taxon names reflect morphological identification and, where available, the closest COI barcode matches.
